# Annotations

**Published:** 1891-04-18

**Authors:** 


### Annotations.

**Lord Hartington and the Derby Hospital.**

The managers of the County Hospital at Derby, after much deliberation and seeking of good advice, have decided to pull down their old hospital and to build a new one. They are to be congratulated on their decision. It was hardly likely after the painful experience of last year and some previous years, and the facts brought to light by the thorough investigation to which the old hospital was subjected, that any attempt would be made to achieve the impossible by trying to restore the old building to sanitary excellence. The Marquis of Hartington, presiding at the meeting for the inauguration of the building fund last week, rightly claimed that the most fundamental of all conditions in the circumstances of a hospital was sanitary excellence. Ancient churches, ancient palaces and many other public and private buildings may claim to be preserved on the ground of their beauty or of their venerable associations. But an ancient hospital is generally a venerable danger, and demands to be removed. The Derby authorities have resolved to spend something over £60,000; a very considerable sum, though by no means too much, if indeed it ultimately proves to be sufficient. There is no need to fear, however, that a committee which has had the courage to face so considerable an expenditure as £60,000 will "spoil the ship for a ha'porth of tar." At the meeting over which Lord Hartington presided, it was announced that more than £32,000 had already been promised or subscribed. This is a noble beginning. The working men of the town and county of Derby are making great efforts to do their share in the enterprise. The more money they contribute the more honour will they confer upon themselves, and the more likely will they be to secure a hospital worthy of the times, and adequate to their various necessities.

**A "Specialist" Disturbance at Cheltenham.**

Readers of The Hospital are well aware that we do not attach an undue value to specialists and specialism in medicine. Nevertheless, specialism there must be if the public are to be able to secure the greatest possible advantages which modern science has to offer. Our contention is, that specialism in medicine is not to be conducted as a profession by itself, but as one of the departments of general medicine and surgery. As a rule, therefore, the proper place for any particular specialism is not a special hospital, but a special department of a general hospital. We may say, in a word, that three main advantages are thus secured: First, economy of management; secondly, concentration of hospital teaching power; and thirdly, the greatest possible efficiency in treatment. These points we do not stop now to argue. They are merely named because of their bearing upon the present position of affairs at Cheltenham. At that town a proposal has been made to create a special department for the treatment of diseases of the ear, throat, and nose, and the wish of the proposers has been that Mr. Griffiths should receive the first appointment. The proposal has, however, been defeated, and a correspondent, writing to the *Cheltenham Examiner*, expresses great satisfaction at the defeat, and bases a general disapproval of the proposal on the ground that there is already at Cheltenham an "eye, ear, and throat" infirmary, which does good work. This infirmary, it seems, was partly originated by Dr. F. A. A. Smith, and has hitherto been to a large extent, if not exclusively, under his care and control. Now, we have nothing whatever to say against Dr. Smith and the infirmary with which he is connected. It is an established institution which, so far as we know, does good work and receives efficient support. It may, therefore, very properly claim the right to exist, and to continue its activities. But to bring forward the fact of the existence of such an institution as a reason why a large general hospital should only be partially equipped for its work is decidedly absurd. The Cheltenham Hospital contains eighty-two beds; in 1889 it treated nearly a thousand in-patients, and over seven thousand out-patients; its annual income is between five and six thousand pounds. It is manifest on the very face of it that a hospital of this magnitude, which is to a large extent a county hospital, should treat every kind of disease incidental to man. There are a very few diseases and organs which can only be properly treated by specialists, and therefore the Cheltenham Hospital, like every other hospital of similar size, should have departments for all the legitimate specialisms; and each one of these departments should have its specialist head. We have no means of knowing on what ground the Committee declined to appoint Mr. Griffiths. If he was a suitable and competent man, and the only candidate in the field, they ought to have appointed him. We assume that there is now no special department at the Cheltenham Hospital for Diseases of the Throat and Ear, and a reference to the *Hospital Annual* for 1890 confirms this assumption. But we also assume that the Committee are anxious to do their duty by the institution, and to bring up its resources to the level of the necessities of the times. If that be so, one of their first duties is to create departments for all the leading specialisms. No local circumstances of any kind can justify the managers of a general hospital in depriving it of any single resource which is necessary to its completeness, as a place of resort for all classes of sick persons, and as a centre of medical example and instruction for all classes of practitioners.

**The Older Nurses.**

A friend of the writer's who takes a great interest in nurses recently had occasion to employ one of them. His family consists of several children and half a-dozen servants; a somewhat formidable household for a nurse to enter. It happened that the particular nurse employed belonged to the older order; not, of course, to the "Gamps," but to the class of trained nurses who are no longer young. The head of the household paid a good deal of critical attention to the way in which this middle-aged woman went about her work. In summing up her professional capacity at the close of her term of work, he found that she had been thoughtful, intelligent, attentive, kind, and generally efficient in a very high degree. She had in fact proved to be, what every really capable trained nurse is, an invaluable helper in time of need. This result came upon the observer with something of a surprise. He had somehow come to think that the younger nurses, with their longer training and more ambitious pretensions, were the flower of the profession, and he was somewhat surprised to find that this particular specimen of the older ones, if not exactly a "flower," was a very excellent "fruit," and as much superior to the average young nurse as the fruit generally is to the flower. This experience put him upon thinking out the subject, and the making of further observations. He has come to the conclusion that the older nurses—those above thirty-five years of age—are very decidedly the best. He, indeed, goes so far as to say that young nurses, meaning by "young" those who are under thirty-five, should as a rule serve only in hospitals and other similar institutions, where they can be properly supervised by Matrons and Sisters. Such young nurses, he considers, are seldom fit for the freedom and individual responsibility which appertain to private nursing. This conclusion entirely coincides with universal experience. To take the medical profession as our most familiar example, we find that the younger men are disposed to think themselves superior to their elders, and very ready to assume that they know all that is to be known. But, as a matter of fact, when such young men come to the bedside, they are simply nowhere compared with really experienced practitioners. Young doctors often practise as consulting physicians, and very sorry specimens of the consultant's capacity they make. The really experienced and capable general practitioner is worth a whole college of such youthful consultants; and nobody knows this better than a physician of mature years. What is true of doctors is equally true of nurses. The ideal nurse must be, above all things, a woman of sense, and this she can seldom be until she has had some experience of life. She must also have practical aptitudes for dealing with all kinds of persons and cases. These also are the fruits of experience. There is no blame to young nurses in asserting them to be less valuable than the older ones. They, also, may do good service; but it is best for them to do it under the direction of their seniors. We are glad to find that middle-aged and older nurses are generally appreciated. It would be a very extraordinary thing indeed if nursing were to prove the one profession in the world in which experience was of no value, and immature youth was more efficient than mature age.

